# Crackle Pitch Rises Progressively during Inspiration in Pneumonia, CHF, and IPF Patients

**DOI:** 10.1155/2012/240160

**Published:** 2012-03-15

**Authors:** Andrey Vyshedskiy, Raymond Murphy

**Affiliations:** Brigham and Women's/Faulkner Hospitals Boston, MA 02130, USA

## Abstract

*Objective*. It is generally accepted that crackles are due to sudden opening of airways and that larger airways produce crackles of lower pitch than smaller airways do. As larger airways are likely to open earlier in inspiration than smaller airways and the reverse is likely to be true in expiration, we studied crackle pitch as a function of crackle timing in inspiration and expiration. Our goal was to see if the measurement of crackle pitch was consistent with this theory. *Methods*. Patients with a significant number of crackles were examined using a multichannel lung sound analyzer. These patients included 34 with pneumonia, 38 with heart failure, and 28 with interstitial fibrosis. *Results*. Crackle pitch progressively increased during inspirations in 79% of all patients. In these patients crackle pitch increased by approximately 40 Hz from the early to midinspiration and by another 40 Hz from mid to late-inspiration. In 10% of patients, crackle pitch did not change and in 11% of patients crackle pitch decreased. During expiration crackle pitch progressively decreased in 72% of patients and did not change in 28% of patients. *Conclusion*. In the majority of patients, we observed progressive crackle pitch increase during inspiration and decrease during expiration. Increased crackle pitch at larger lung volumes is likely a result of recruitment of smaller diameter airways. An alternate explanation is that crackle pitch may be influenced by airway tension that increases at greater lung volume. In any case improved understanding of the mechanism of production of these common lung sounds may help improve our understanding of pathophysiology of these disorders.

## 1. Introduction

Crackles are intermittent short-lived sounds that emanate from the lung and are associated with pulmonary disorders including pneumonia (PN), congestive heart failure (CHF), and interstitial pulmonary fibrosis (IPF) [1–3]. The mechanism underlying crackle generation is not well understood, however, and the spectral, temporal, and spatial characteristics of crackles have not been well quantified. In this paper we characterized crackles in patients with PN, CHF, and IPF. We quantified these events using multiple microphones placed on the chest surface and we focused in particular on differences in pitch between crackles generated at different lung volumes.

## 2. Materials and Methods

### 2.1. Patients Selection

Patients were selected for this study from a pool of patients who had undergone lung sound analysis as a part of a broader study of the correlation of disease processes with lung sounds patterns. To select patients into this study, we identified hospitalized and outpatients of a community teaching hospital who were diagnosed as having a specific cardiopulmonary disease or were considered to be normal by their caregivers. These patients were drawn from more than one thousand patients on whom we have both the diagnosis and the lung sound analysis. The diagnostic category of each of the patients was that of the clinicians caring for these patients. This was reviewed by the senior author to be sure they were consistent with established criteria.

Subjects were examined with a 16-channel lung sound analyzer (Stethographics Model STG1602). The STG was described in detail in Bergstresser et al. and in Murphy et al. [4–6]. In short, the STG uses electret microphones mounted in stethoscope chest pieces to record data on a PC. Fourteen microphones are incorporated into a soft foam microphone pad. The microphone pad is positioned on a stretcher or a plastic reclining chair positioned at a 45 degree angle. In this study, subjects were instructed to lie in a recumbent position on the microphone pad. Subjects were instructed to breathe more deeply than normal. Typically 3 to 6 full breaths were captured in a 20-second recording.

Crackles were defined in accordance with accepted criteria [7, 8]. Coarse crackles are defined as discontinuous sounds with pitch less than 400 Hz. Fine crackles are defined as discontinuous sounds with pitch greater than 400 Hz [9]. The STG software automatically identified crackles in all full breaths. The validation of the use of the device as a crackle counter has been reported [10]. The goal of this study was to compare crackle pitch between crackles generated during early, mid, and late-inspiration and separately between crackles generated during early, mid, and late-expiration. Accordingly, to study crackle pitch changes during inspiration, we only accepted those patients who had two or more crackles in each interval: early, mid, and late-inspiration. These patients included 34 with pneumonia, 38 with heart failure, and 28 with interstitial fibrosis. A single 20-second recording contained 3 or more breathing cycles. Therefore, patients accepted into this part of the study had at least 6 early, 6 mid, and 6 late-inspiratory crackles in the 20-second recording.

 To study crackle pitch changes during expiration, we only accepted those patient who had two or more crackles in each interval: early, mid, and late-expiration. These patients included 10 patients with pneumonia and 8 patients with interstitial fibrosis. No patients with heart failure had the minimum required number of expiratory crackles.

### 2.2. Crackle Family Analysis

The concept of a crackle family was introduced and validated in Vyshedskiy et al. [11]. In short, a single crackle event can be detected by multiple microphones located on the chest surface. The group of waveforms corresponding to the single crackle event and recorded by multiple microphones is referred to as a crackle family. The channel with highest crackle amplitude is called the mother crackle and the corresponding deflections at other channels are called daughter crackles. These definitions are consistent with the theory that the event, which generated a crackling sound, had occurred closer to the mother channel microphone than to the other microphones. Indeed evidence supporting this theory can be found by examination of the stack plots of the crackles [11].

In this study, crackle pitch of the mother crackle was used for analysis. Analysis of the mother crackle started by identification of its highest deflection. The half period to the left of the highest peak is referred as T1. The half period to the right of the highest peak is marked as T2. Crackle pitch is calculated from 4 consecutive half periods, with T1 as the 1st half period.

The study was approved by the Institutional Review Board of the Faulkner Hospital.

## 3. Results

**Figure 1:**
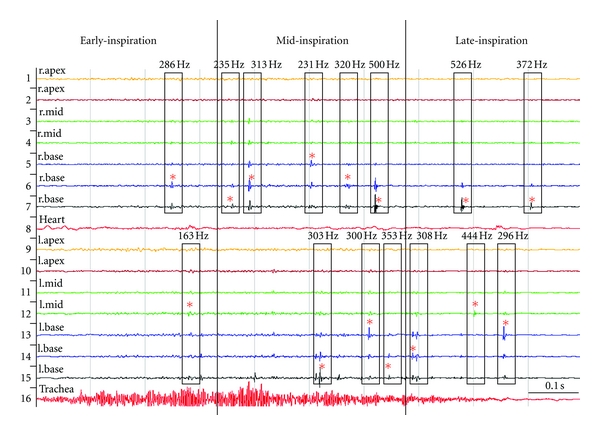
A time amplitude plot of inspiratory lung sounds recorded from a patient with pneumonia. Channels 1 to 7 were recorded from the right lung, channels 9 to 15 were recorded from the left lung, channel 8 was recorded from the heart (not used for crackle analysis), and channel 16 was recorded from the trachea (not used for crackle analysis). A black border indicates crackle families, mother crackles are marked by a star “*”, crackle pitch is shown on top of each border. Thick vertical lines indicate the boundaries between early, mid-, and late-inspiration.

In a time-amplitude plot crackles look like spikes.  Figure 1 shows lung sounds recorded during a single inspiration from a patient with pneumonia. The stack plot shows 16 channels recorded from the patient's back. The automated computer algorithm identified 2 early, 8 mid, and 5 late-inspiratory crackles. Seven breaths were recorded in this patient in 20 seconds. The average crackle rate was 2.0 crackles per breath in early, 7.6 in mid, and 7.7 in late-inspiration. While individual crackles vary in their pitch, there is a clear trend toward progressive increase in crackle pitch during inspiration: the average crackle pitch in this patient was 269 Hz in early, 317 Hz in mid, and 376 Hz in late-inspiration.

The progressive increase in crackle pitch during inspiration was observed in the majority of patients.  Table 1 shows the number of patients in each group and in each disease. A reverse phenomenon was observed during expiration. The progressive decrease of crackle pitch was observed during expiration in most patients, Table 2.

 In patients whose crackle pitch increased during inspiration, the average increase was around 40 Hz, Table 3.

In patients whose crackle pitch decreased during expiration, the average decrease was also around 40 Hz, Table 4.

## 4. Discussion

There is considerable evidence, summarized by Forgacs [1], that inspiratory crackles are caused by airway opening. There is also some evidence that expiratory crackles are caused by abrupt airway closing [2, 3]. Our group recently reported that crackle rate and crackle pitch do not vary significantly from breath to breath [12]. Even when breaths were separated by cough and vital capacity maneuver crackle rate and crackle pitch did not vary significantly in a single auscultation session. In this report, we systematically examined crackle pitch as a function of crackle timing during a single breath. We have observed that in the majority of patients crackle pitch progressively increases during inspiration and progressively decreases during expiration. This finding is consistent with the clinical observations that coarser lower-pitched crackles tend to occur in early inspiration and that finer higher-pitched crackles tend to occur in late-inspiration.

Several hypotheses are consistent with this observation. The first hypothesis proposes that crackle pitch is determined by airway diameter. Airways with progressively smaller diameter are recruited during inspiration. Therefore, crackles recorded during the beginning of inspiration are expected to be generated by larger diameter airways than the crackles generated at the end of inspiration. If crackle pitch is determined by airway diameter then crackles generated in the beginning of inspiration are expected to have lower pitch than the crackles generated at the end of inspiration. The progressive pitch decrease during expiration is explained similarly as smaller airways close earlier in expiration than larger airways.

How can we explain the association between crackle pitch and the airway diameter? When an airway collapses, the walls of the airway become flat. In this position, the airway wall tissue can be compared to a guitar string fixed at both ends. Airways opening during an inspiration sets airway walls tissue into motion. The resonant frequency of a guitar string is determined by its length, tension, and density. Shorter lengths, higher tension, and lower density increase the resonant frequency. If the analogy with a guitar string has scientific merits, we can expect higher-pitched crackles generated by smaller airways with higher tension and lower airway wall density.

The second hypothesis uses airway tension independent of airway size to explain changes in crackle pitch in a fashion similar to the effect of tension on a string's resonant frequency. If crackle pitch is influenced by airway tension, then greater crackle pitch can be explained by increased airway tension at greater lung volume.

Finally, the third hypothesis proposes that crackles are generated closer to the chest wall at larger lung volumes. According to this hypothesis, crackles generated at lower lung volume are generated deeper in the lung and are low pass filtered by the lung parenchyma to a greater extend. This hypothesis concludes that the difference in pitch is the result of the difference of sound filtering by lung parenchyma. It is also feasible that all three mechanisms contribute to the increase of crackle pitch at greater lung volume.

In summary, we observed that within a single breath crackle pitch tends to increase during inspiration and decrease during expiration. While the clinical implications of this observation are not clear, better general understanding of the mechanism of crackles production offers the promise of improving noninvasive diagnosis of lung disorders.

## Figures and Tables

**Table 1:** Number of patients whose crackle pitch increased, decreased and did not change during inspiration.

	Pneumonia	CHF	Interstitial fibrosis	Total
Increased	28	28	23	79 (79%)
No change	2	6	2	10 (10%)
Decreased	4	4	3	11 (11%)

**Table 2:** Number of patients whose crackle pitch increased, decreased, and did not change during expiration.

	Pneumonia	CHF	Interstitial fibrosis	Total
Increased	0	0	0	0 (0%)
No change	3	0	2	5 (28%)
Decreased	7	0	6	13 (72%)

**Table 3:** An average change in crackle pitch among the patients whose crackle pitch increased during inspiration.

	Pneumonia	CHF	Interstitial fibrosis	Total
From early to mid-inspiration (Hz)	46 ± 47	43 ± 55	40 ± 46	42 ± 49
From mid to late-inspiration (Hz)	49 ± 45	57 ± 45	38 ± 41	48 ± 44

**Table 4:** An average change in crackle pitch among the patients whose crackle pitch decreased during expiration.

	Pneumonia	CHF	Interstitial fibrosis	Total
From early to mid-expiration (Hz)	74 ± 40	N/A	17 ± 17	53 ± 43
From mid to late-expiration (Hz)	37 ± 48	N/A	49 ± 15	43 ± 36

## Abbreviations

CHF: Congestive Heart Failure
IPF: Interstitial Pulmonary Fibrosis
PN: Pneumonia.

## References

[1] Forgacs P (1967). Crackles and wheezes. *Lancet*.

[2] Fredberg JJ, Holford SK (1983). Discrete lung sounds: crackles (rales) as stress-relaxation quadrupoles. *Journal of the Acoustical Society of America*.

[3] Vyshedskiy A, Alhashem RM, Paciej R (2009). Mechanism of inspiratory and expiratory crackles. *Chest*.

[4] Bergstresser T, Ofengeim D, Vyshedskiy A, Shane J, Murphy R (2002). Sound transmission in the lung as a function of lung volume. *Journal of Applied Physiology*.

[5] Murphy RL, Vyshedskiy A, Power-Charnitsky VA (2004). Automated lung sound analysis in patients with pneumonia. *Respiratory Care*.

[6] Murphy RLH (2008). In defense of the stethoscope. *Respiratory Care*.

[7] Murphy RLH, Holford SK, Knowler WC (1977). Visual lung sound characterization by time expanded wave form analysis. *The New England Journal of Medicine*.

[8] Sovijarvi AHA, Vanderschoot J, Earis JE (2000). Computerized Respiratory Sound Analysis (CORSA) recommended standards for terms and techniques. *European Respiratory Review*.

[9] (1977). *American Thoracic Society Committee on Pulmonary Nomenclature*.

[10] Murphy RLH, Del Bono EA, Davidson F (1989). Validation of an automatic crackle (Rale) counter. *American Review of Respiratory Disease*.

[11] Vyshedskiy A, Bezares F, Paciej R, Ebril M, Shane J, Murphy R (2005). Transmission of crackles in patients with interstitial pulmonary fibrosis, congestive heart failure, and pneumonia. *Chest*.

[12] Vyshedskiy A, Ishikawa S, Murphy R (2011). Crackle pitch and rate do not vary significantly during a single examining session in patients with pneumonia, congestive heart failure, and interstitial pulmonary fibrosis. *Respiratory Care*.

